# HTSSIP: An R package for analysis of high throughput sequencing data from nucleic acid stable isotope probing (SIP) experiments

**DOI:** 10.1371/journal.pone.0189616

**Published:** 2018-01-03

**Authors:** Nicholas D. Youngblut, Samuel E. Barnett, Daniel H. Buckley

**Affiliations:** School of Integrative Plant Science, Cornell University, Ithaca, New York, United States of America; Oak Ridge National Laboratory, UNITED STATES

## Abstract

Combining high throughput sequencing with stable isotope probing (HTS-SIP) is a powerful method for mapping *in situ* metabolic processes to thousands of microbial taxa. However, accurately mapping metabolic processes to taxa is complex and challenging. Multiple HTS-SIP data analysis methods have been developed, including high-resolution stable isotope probing (HR-SIP), multi-window high-resolution stable isotope probing (MW-HR-SIP), quantitative stable isotope probing (qSIP), and ΔBD. Currently, there is no publicly available software designed specifically for analyzing HTS-SIP data. To address this shortfall, we have developed the *HTSSIP* R package, an open-source, cross-platform toolset for conducting HTS-SIP analyses in a straightforward and easily reproducible manner. The *HTSSIP* package, along with full documentation and examples, is available from CRAN at https://cran.r-project.org/web/packages/HTSSIP/index.html and Github at https://github.com/buckleylab/HTSSIP.

## Introduction

Stable isotope probing of nucleic acids (DNA- and RNA-SIP) is a powerful method for mapping *in situ* metabolic processes, such as nitrogen and carbon cycling, to microbial taxa. Historically, the sensitivity of nucleic acid SIP has been limited by the low throughput of DNA sequencing and the low taxonomic resolution of DNA fingerprinting techniques [1,2]. Recently, DNA- and RNA-SIP have been combined with high throughput sequencing of PCR amplicons (HTS-SIP), which allows researchers to map *in situ* metabolic processes to thousands of taxa resolved at a fine taxonomic resolution [3–5].

While HTS-SIP is proving to be a very useful method for exploring *in situ* metabolic processes in complex microbial communities, the accurate analysis of HTS-SIP datasets is complex [6,7]. Multiple strategies have been developed for analyzing HTS-SIP data, including high-resolution stable isotope probing (HR-SIP) [5], multi-window high-resolution stable isotope probing (MW-HR-SIP) [7], quantitative stable isotope probing (qSIP) [3], and ΔBD (delta Buoyant Density) [5]. Prior to the development of these HTS-SIP analysis methods, DNA- and RNA-SIP experiments that utilized Sanger or high throughput sequencing were usually analyzed with standard statistical processes (*e*.*g*. t-tests), which have low sensitivity and/or high false positive rates when applied to sequence data [7,8]. The goals of HTS-SIP analysis methods differ, with HR-SIP and MW-HR-SIP designed to accurately identify taxa that have incorporated isotopically labeled substrate (*i*.*e*. ‘incorporators’), while the main goal of qSIP and ΔBD is to quantify the amount of isotopic enrichment for each taxon (*i*.*e*. atom % excess). While all methods use amplicon sequences (*e*.*g*. 16S rRNA or fungal ITS sequences) from multiple fractions of each isopycnic gradient, HR-SIP, MW-HR-SIP, and ΔBD solely use sequence data while qSIP additionally requires qPCR derived estimations of gene copy number from each gradient fraction. Recently, Youngblut and Buckley developed a HTS-SIP simulation model and showed that MW-HR-SIP is more accurate for identifying incorporators than HR-SIP and qSIP, while qSIP is generally more precise than ΔBD for quantifying isotopic enrichment [7].

Currently, there are no publicly available software tools specifically designed for HTS-SIP analysis, which forces researchers to either recreate the analysis methods described in existing HTS-SIP studies or develop ad-hoc analysis methods. Both of these options decrease the ease of use and reproducibility of HTS-SIP analyses. To address this deficiency, we developed the *HTSSIP* R package, which includes the following features that aid in HTS-SIP analyses:

- Functions for conducting HR-SIP, MW-HR-SIP, qSIP, and ΔBD to analyze data from DNA-SIP and RNA-SIP experiments, plus functions for conducting historically used statistical methods (*e*.*g*. t-tests comparing taxon abundance in “heavy” fractions).
- Functions for performing HTS-SIP dataset simulation, as described [7]
- Functions for exploratory analysis of simulated HTS-SIP data, useful for predicting how different experimental designs can alter experimental outcomes
- Functions for exploratory analysis of real HTS-SIP data, useful for conducting post-hoc analyses
- Ability to run analyses with parallel processing
- Extensive documentation and tutorials (see the *HTSSIP* package vignettes)

## Package description

### Input data

**Fig 1:**
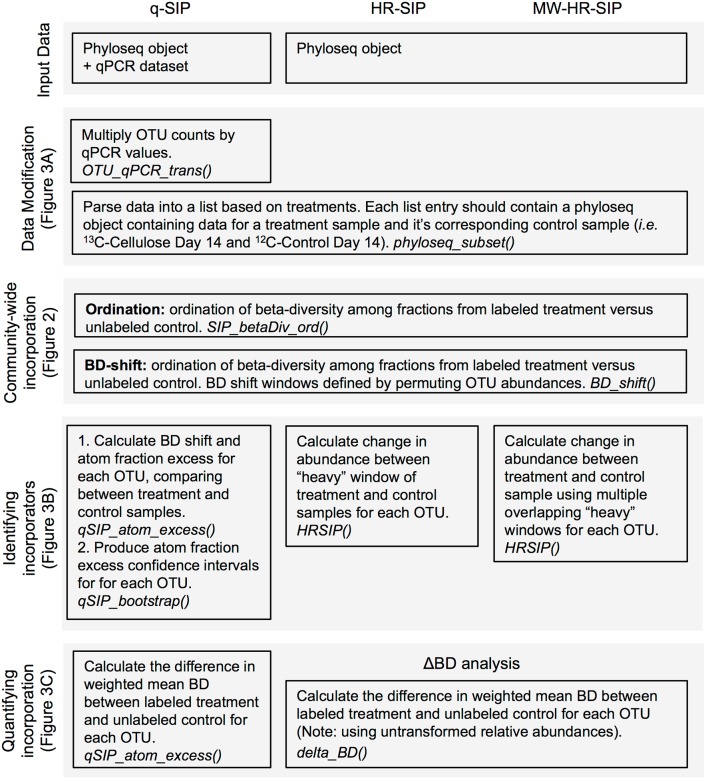
*A diagram depicting the possible HTS-SIP analyses available in the* HTSSIP *R package*. The R functions to conduct each workflow step are italicized, and the figure references refer to example data produced by these workflow steps.

Dataset input is handled by the *Phyloseq* R package, a feature-rich package for general microbiome data analysis that can be used to import many common microbiome data formats [9]. *HTSSIP* includes convenience functions to easily and flexibly designate the experimental design of the SIP experiment for downstream HTS-SIP analyses (Fig 1).

### HTS-SIP dataset exploratory analyses

A common first step in analyzing nucleic acid SIP data is to quantify the total nucleic acid concentration or gene copy number (estimated by qPCR) across density gradients in order to determine the buoyant density (BD) “shift” of nucleic acids in isotopically labeled treatments versus unlabeled controls [10,11]. The general expectation is that a “shift” of nucleic acid BD from “light” towards “heavy” densities is indicative of isotope incorporation. However, in a well designed SIP experiment, the ratio of added to endogenous substrate should be small, and this can produce an imperceptible BD shift [4]. In addition, an extensive shift may indicate excessive cross-feeding [12]. HTS-SIP methods can detect taxa that have incorporated low levels of isotope, or occur at frequencies that are so low that they do not cause a shift in the overall BD of community nucleic acids [5]. As a result, analysis of the BD distribution of total nucleic acids within density gradients is of little utility in assessing the results of nucleic acid SIP experiments performed on complex communities.

As a simpler alternative, which leverages the power of high-throughput sequencing techniques, BD “shifts” can be inferred solely from sequence data [4,5]. Given that incorporators will be more abundant in “heavy” gradient fractions of the labeled treatment versus the unlabeled control, a BD shift can be inferred by assessing the beta-diversity between treatment and control gradient fractions. This approach is more sensitive for detecting community-level isotope incorporation than the approach of quantifying total nucleic acid concentration across the density gradient [7]. The *HTSSIP* R package implements two methods for using beta-diversity to assess isotope incorporation at the community-level: an ordination approach and an approach that expresses beta-diversity between corresponding treatment and control fractions as a function of their BD (Fig 1).

**Fig 2:**
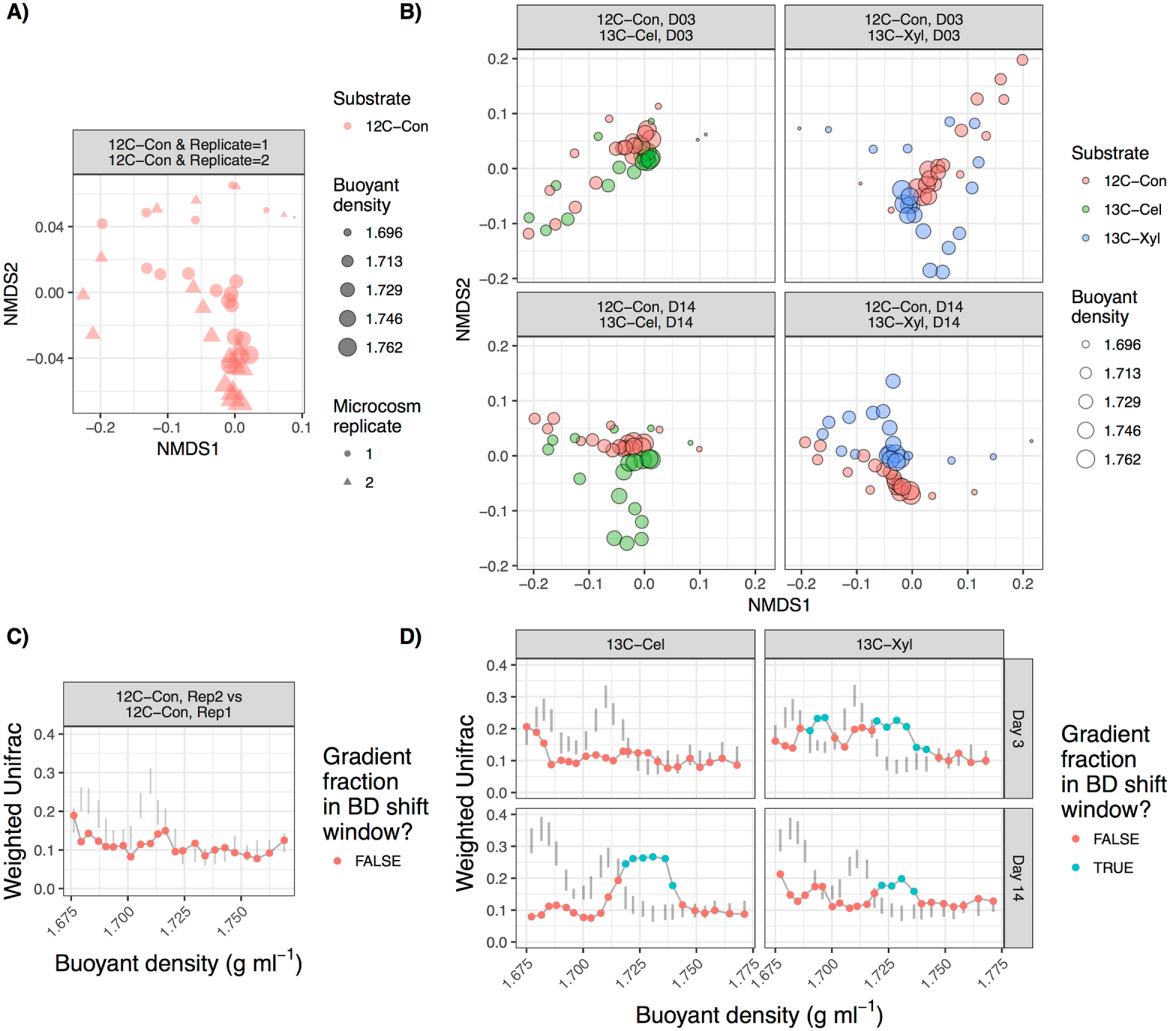
*Examples of analyses available in the* HTSSIP *R package for visualizing results from HTS-SIP experiments*. These figures were generated using data from an HTS-SIP experiment that consisted of 3 treatments where only the isotopic label was varied: an unlabled control (12C-Con), ^13^C-xylose (13C-Xyl), and ^13^C-cellulose (13C-Cel) [7]. DNA was extraced from each treatment 3 and 14 days after substrate addition, subjected to CsCl fractionation, and 16S rRNA sequencing was performed on ~24 fractions per CsCl gradient. Analyzing beta-diversity between gradient fractions can reveal changes in the buoyant density (BD) distribution of DNA in ^13^C-labeled treatments versus the unlabeled control. Panel A is a non-metric multidimensional scaling (NMDS) ordination comparing beta-diversity (weighted Unifrac) between gradient fractions from two unlabeled replicates, which indicates that sequence composition varies greatly with BD but not between experimental replicates. Panel B is an NMDS ordination similar to Panel A, but each facet contains a ^13^C treatment and the corresponding unlabeled control. Large relative distance between “heavy” control and labeled gradient fractions indicates a change in community composition caused by shift in DNA BD that resulted from ^13^C isotope incorporation. The NMDS stress values ranged from 0.06 to 0.07. Panels C and D depict the same data as in Panels A and B, respectively, but only depict beta-diversity among gradient fractions that correspond in BD between labeled treatment and unlabeled control fractions. In this visualization, increases in beta-diversity indicate a change in community composition caused by a shift in DNA BD resulting from ^13^C isotope incorporation. The line ranges represent 95% confidence intervals (CI) calculated by permuting OTU abundances and recalculating beta-diversity (100 bootstrap replicates), and actual beta-diversity values are represented by colored circles. "BD shift windows" (blue circles) indicate regions defined by ≥3 consecutive fractions with beta-diversities greater than expected by chance (*i*.*e*. exceeding the CI).

The ordination approach simply involves pairwise calculations of a beta-diversity metric between all gradient fractions from isotopically labeled treatments and corresponding unlabeled controls, followed by visualizing the distance matrix with either principal coordinates analysis (PCoA) or non-metric multidimensional scaling (NMDS). An increase in beta-diversity between corresponding gradient fractions of labeled samples and controls is expected if isotope incorporation causes a change in the BD of OTUs (Fig 2A & 2B).

While the ordination approach provides a useful overview of community-wide isotope incorporation, the extent of incorporation is difficult to compare among multiple treatments (*e*.*g*. ^13^C-cellulose versus ^13^C-xylose). The second approach implemented in the *HTSSIP* package visualizes DNA BD shifts by calculating pairwise beta-diversity of corresponding gradient fractions between treatment and control gradients (or control versus control to assess technical variation among replicates). To deal with partially overlapping gradient fractions between gradients, the weighted mean beta-diversity is calculated from all treatment gradient fractions that overlap each control gradient fraction, with weights defined as the percent overlap in the BD range of each fraction (Fig 2C & 2D). A permutation test is used to identify BD ranges of high beta-diversity resulting from BD shifts (“BD shift windows”). The permutation test involves constructing bootstrap confidence intervals (CI) of beta-diversity by permuting OTU abundances among control fractions to generate a null model “treatment” that is compared against the actual control fractions. This null model represents the baseline beta-diversity expected if the treatment was just a randomly shuffled version of the control. A note in interpreting these data is that isotope incorporation will cause DNA to shift out of “light” gradient fractions and into “heavy” gradient fractions. Hence, in the presence of isotope incorporation, high beta-diversity can be observed in both “heavy” and “light” gradient fractions. Alternatively, in the absence of isotope incorporation, beta-diversity will remain low across all gradient fractions.

### Identifying incorporators

**Fig 3:**
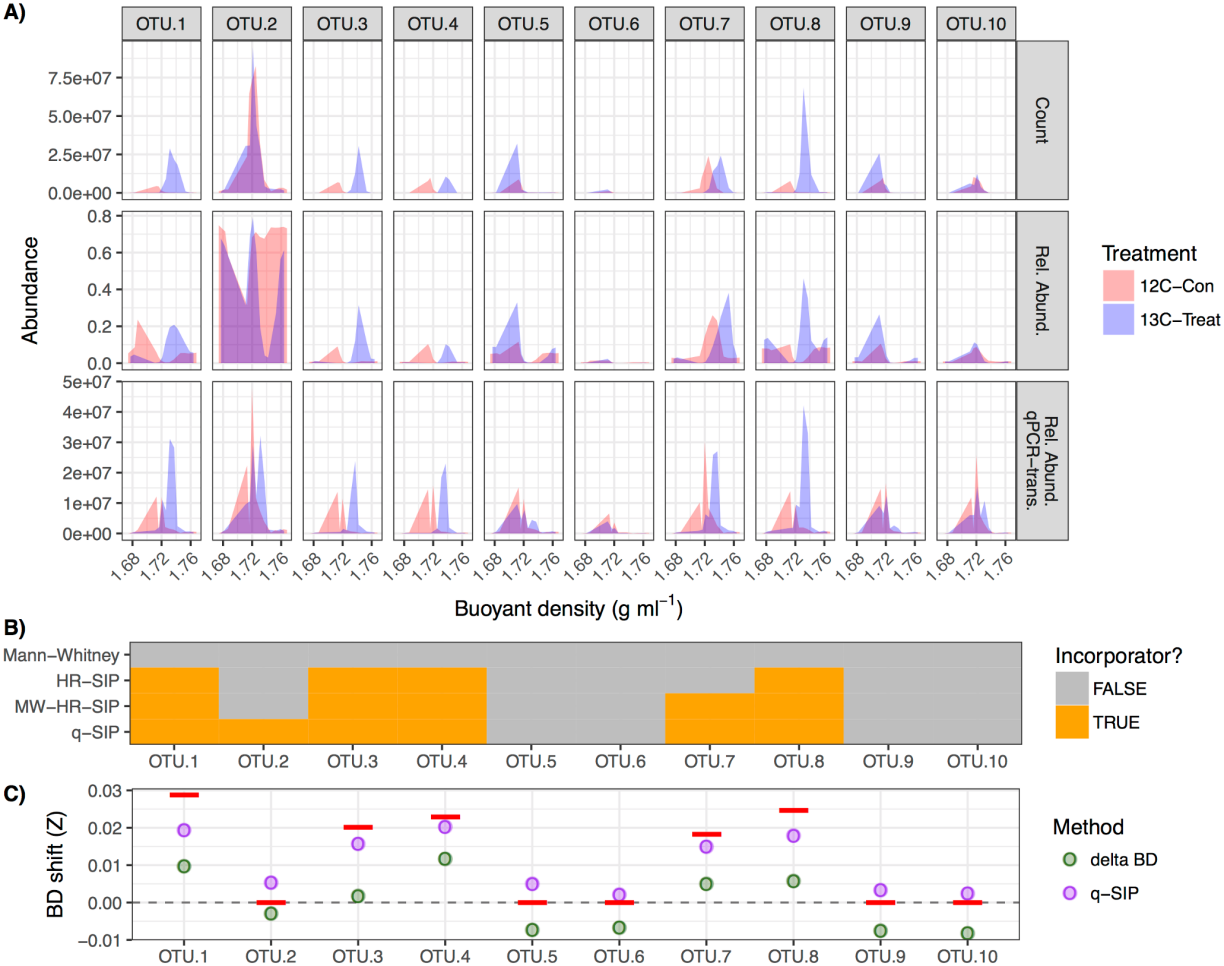
*Examples of using the* HTSSIP *R package for data processing*, *data exploration*, *incorporator identification*, *and quantification of BD shifts*. The SIPSim toolset was used to simulate a simple HTSSIP dataset consisting of two treatments: a ^13^C-treatment (“13C-Treat”) and a ^12^C-control (“12C-Con”). Each treatment has 3 replicates, with each consisting of 24 gradient fractions [7]. Half of the ten OTUs (OTU 1, 3, 4, 7, and 8) were randomly assigned an atom excess ^13^C between 30 and 100%. [3]. Panel A depicts the raw abundances (“Counts”), fractional relative abundance (“Rel. Abund.”), and relative abundances transformed by simulated qPCR data (“Rel. Abund. qPCR-trans.”). For clarity, only 1 of the 3 experimental replicates is shown. Panel B shows those OTUs identified as “incorporators” (incorporated ^13^C into genomic DNA) by HR-SIP, MW-HR-SIP, qSIP, or by using a “heavy-SIP” approach (the Mann Whitney U test). Panel C shows the BD shift of each OTU as determined by ΔBD or qSIP. The dashed line signifies a BD shift (Z) of 0.0 g ml^-1^, and the red bars show the true theoretical BD shift resulting from ^13^C isotope incorporation.

HR-SIP, MW-HR-SIP, and qSIP can all be used to identify incorporators. To illustrate the application of HR-SIP, MW-HR-SIP, and qSIP in the *HTSSIP* R package, we simulated a simplified HTS-SIP dataset consisting of 10 OTUs (Fig 3A). Our purpose here is merely to illustrate functions of the *HTSSIP* R package; comprehensive assessment of the accuracy of these techniques (and historically used DNA- and RNA-SIP analysis methods) is available elsewhere [7]. Briefly, HR-SIP identifies incorporators by utilizing DESeq2 to identify OTUs that have high differential relative abundance in “heavy” fractions of labeled treatment versus unlabeled control [8]. MW-HR-SIP takes the same relative abundance based approach as HR-SIP but uses multiple overlapping “heavy” BD windows (while correcting for multiple hypotheses). In contrast, qSIP uses qPCR data to transform OTU relative abundance distributions into pseudo-absolute abundance distributions (Fig 3A), and then BD shifts are determined from these transformed distributions by calculating the difference in center of mass for each OTU in treatment versus control gradients. Atom fraction excess can thus be calculated for specific isotopes (*e*.*g*. ^13^C or ^15^N) based on the calculations described in the work of Hungate and colleagues [3]. In order to identify incorporators, a permutation test is used to construct bootstrap confidence intervals of atom fraction excess. The *HTSSIP* package also provides functions for conducting historically used DNA- and RNA-SIP analysis methods (*e*.*g*. using t-tests to compare OTU abundances between “heavy” control versus treatment fractions). We refer to these methods collectively as “heavy-SIP”, which have been extensively compared to HR-SIP methods in other work [7]. Sensitivity in identifying incorporators can depend on the methods used (Fig 3B), and although we only provide a simple comparison of methods here, the senstivity and specifity of these results reflect those of Youngblut and Buckley [7]. SIP experiments can be simulated using the SIPSim toolset [7], and these data analyzed using the *HTS-SIP* R package. Such *in silico* evaluation is valuable for predicting possible experimental outcomes and the expected analytical accuracy of SIP experiments based on details of experimental design prior to conducting experiments.

The *HTSSIP* package implements HR-SIP based on the code provided in the work of Pepe-Ranney and colleagues [5]. MW-HR-SIP is implemented in *HTSSIP* based on the R code provided in the SIPSim HTS-SIP dataset simulation toolset [7]. The *HTSSIP* implementation of qSIP is based on the method’s description in the work of Hungate and colleagues [3]. Implementations of each method include the option for parallel processing of each algorithm. Parallelization is implemented through the *plyr* R package [13], which allows for various parallel backends to be used such as *doSNOW* and *doParallel*.

### Quantifying isotopic enrichment

Unlike HR-SIP and MW-HR-SIP, the main goal of qSIP and ΔBD is to quantify isotopic enrichment. To illustrate the use of the *HTSSIP* package for conducting qSIP and ΔBD, we applied both analyses to the simplified HTS-SIP dataset described above (Fig 3C). ΔBD is implemented in *HTSSIP* as described in the work of Pepe-Ranney and colleagues [5]. As shown in Youngblut and Buckley [7], qSIP and ΔBD can produce substantially different estimates of isotope incorporation.

### Simulating datasets

The *HTSSIP* package provides functions to simulate simple HTS-SIP datasets for use in software testing, analysis pipeline development, and gaining familiarity with software and data formats. However, the SIPSim toolset is recommended for evaluating possible SIP experimental designs and for testing the accuracy of HTS-SIP analyses, because the simulation framework for SIPSim is based on the physics of isopycnic centrifugation, unlike the simulations possible with *HTSSIP* [7]. *HTSSIP* utilizes *coenocliner*, an R package designed for simulating taxon abundance across environmental gradients, to simulate taxon abundance distributions across buoyant density gradient fractions [13].

## Future work

Future development of the *HTSSIP* package will include i) functions for mapping incorporator status to phylogenies and visualizing the results ii) direct integration with the SIPSim toolset for rapid HTS-SIP experimental design and assessment of accuracy iii) functions analyzing shotgun metagenome data derived from SIP experiments.

## Conclusions

Given the power of HTS-SIP for mapping *in situ* metabolism to taxonomic identity, adoption of the technique by researchers will greatly help to resolve connections between microbial ecology and taxonomy. Currently, HTS-SIP data analysis is complex, with few existing computational tools to aid researchers. The R package *HTSSIP* provides a single, standardized analysis pipeline that facilitates reproducible analyses on HTS-SIP datasets and direct cross-study comparisons. Moreover, *HTSSIP* can be combined with the SIPSim toolset to simulate and evaluate possible DNA-SIP experimental designs.
